# Data on novel DNA methylation changes induced by valproic acid in human hepatocytes

**DOI:** 10.1016/j.dib.2017.11.031

**Published:** 2017-11-10

**Authors:** JarnoEJ Wolters, SimoneGJ van Breda, SandraM Claessen, TheoMCM de Kok, JosCS Kleinjans

**Affiliations:** Department of Toxicogenomics, GROW-School for Oncology and Developmental Biology, Maastricht University Medical Center, P.O. Box 616, 6200 MD Maastricht, The Netherlands

**Keywords:** DNA methylation, Methylated DNA Immuno-Precipitation-sequencing (MeDIP-seq), Primary human hepatocytes (PHHs), Steatosis, Valproic acid (VPA)

## Abstract

Valproic acid (VPA) is a widely prescribed antiepileptic drug in the world. Despite its pharmacological importance, it may cause liver toxicity and steatosis. However the exact mechanism of the steatosis formation is unknown. The data presented in this DIB publication is used to further investigate the VPA-induced mechanisms of steatosis by analyzing changes in patterns of methylation. Therefore, primary human hepatocytes (PHHs) were exposed to VPA at a concentration which was shown to cause steatosis without inducing overt cytotoxicity. VPA was administered for 5 days daily to PHHs. Furthermore, after 5 days VPA-treatment parts of the PHHs were followed for a 3 days washout. Differentially methylated DNA regions (DMRs) were identified by using the ‘Methylated DNA Immuno-Precipitation - sequencing’ (MeDIP-seq) method. The data presented in this DIB demonstrate induced steatosis pathways by all DMRs during VPA-treatment, covering interesting drug-induced steatosis genes (persistent DMRs upon terminating VPA treatment and the *EP300* network). This was illustrated in our associated article (Wolters et al., 2017) [1]. MeDIP-seq raw data are available on ArrayExpress (accession number: E-MTAB-4437).

**Specifications Table**

**Table:** 

Subject area	*Biology*
More specific subject area	*(Hepato)toxicogenomics*
Type of data	*Figure and Tables*
How data was acquired	*Illumina HiSeq. 2000 sequencer*
Data format	*Differentially methylated DNA regions/genes, pathways, statistical analysis*
Experimental factors	*Primary human hepatocytes (PHHs) were treated by valproic acid (VPA) at an incubation concentration of 15 mM for 5 days daily followed by a washout of 3 days*
Experimental features	*The treated samples were corrected for time-matched vehicle controls.*
	*The persistent changes were identified by determining DNA methylation similarities between samples of 5 days daily VPA-treatment and samples of 3 days washout upon the 5 days daily VPA-treatment*
Data source location	*Department of Toxicogenomics, Maastricht University, the Netherlands*
Data accessibility	*Methylated DNA Immuno-Precipitation – sequencing (MeDIP-seq) raw data are available on ArrayExpress (accession number: E-MTAB-4437).*

**Value of the Data**

• The data derived from primary human hepatocytes (PHHs) treated with valproic acid (VPA) as well as the data analysis approaches in this publication can serve as a benchmark to investigate the epigenetics effects of other hepatotoxic compounds, since the data show that Methylated DNA Immuno-Precipitation – sequencing (MeDIP-seq) analysis is highly informative in disclosing novel mechanisms of VPA-induced toxicity in PHHs.
• The investigation of persistent methylation changes in PHHs provides a new perspective for other studies related to the drug-induced steatosis or other forms of toxicity.
• The listed gene *EP300* together with the neighbors, of the network analysis, can be used for the development of biomarker screening tools for the early detection of drug-induced steatosis or other forms of toxicity, also by using other cell types.

## 1 Data

**Fig. 1:**
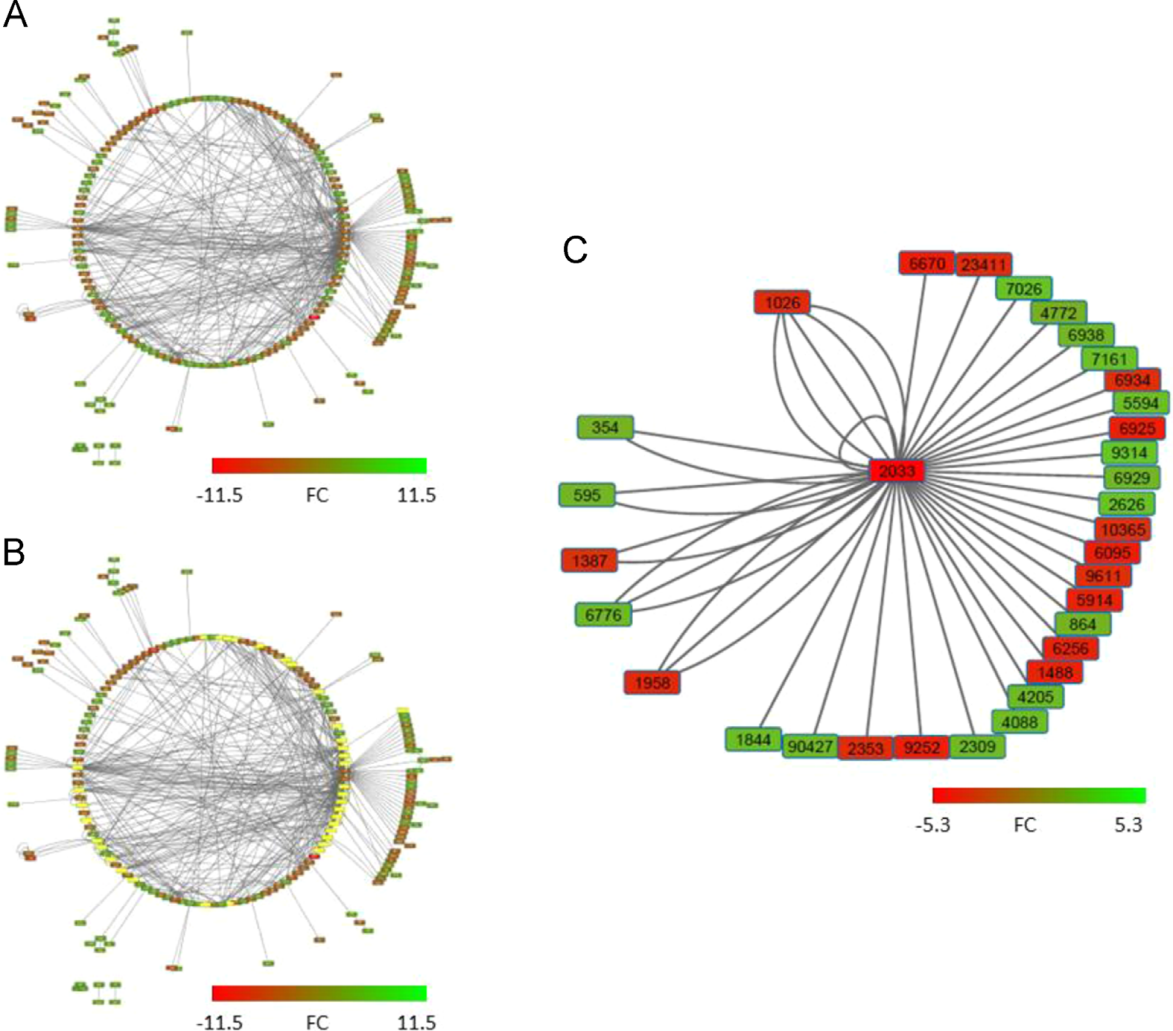
(A, B) Large molecular interaction network identified by ConsensusPathDB, consisting of 201 genes derived from differentially methylated regions in PHHs after 5 days daily VPA-treatment. (C) VPA-induced sub-molecular interaction network of the 33 neighbor-genes of gene 2033 (*EP300*) in PHHs identified by ConsensusPathDB. EntrezGene IDs of the 33 neighbour-genes as well as the Gene symbol, Gene Name and the FCs can be found in Table 3. green = hypermethylation; red = hypomethylation; yellow = neighbors of the gene 2033 (*EP300*) of the large molecular interaction network. PHHs, primary human hepatocytes; VPA, valproic acid; FCs, fold changes.

Methylated DNA Immuno-Precipitation – sequencing (MeDIP-seq) analysis showed that the methylation of more than 6000 genes significantly changed after 5 days daily valproic acid (VPA)-treatment (3006 hypermethylated differentially methylated DNA regions (DMRs) and 3077 hypomethylated DMRs). 31 DMRs were persistently methylated after taking the compound away (11 hypomethylated DMRs and 20 hypermethylated DMRs). The names and functions of those persistent DMRs are shown in Table 1. Furthermore, the 3006 hypermethylated and 3077 hypomethylated DMRs were classified into 119 significantly enriched pathways (Table 2). The unique genes of all those 119 significantly enriched pathways, which have shown significant methylation changes in our data after 5 days daily VPA-treatment, formed a complex network module (Fig. 1A-B). The gene *EP300* has 33 neighbors (Fig. 1B-C) and the gene names, gene symbols, and fold changes (FCs) of those neighbors were shown in Table 3. A more detailed description of those findings can be found in Wolters et al. [1].

**Table 1:** Names and functions of the 20 persistently hypermethylated DMRs annotated to 15 unique Entrez Genes (A) and the 11 hypomethylated DMRs annotated to 9 unique Entrez Genes (B) of the MEDIP-seq analysis after the exposure of PHHs for 5 days daily to VPA followed by 3 days washout. PHHs, primary human hepatocytes; VPA, valproic acid; DMRs, differentially methylated DNA regions.

**A) 20 persistently hypermethylated DMRs annotated to 15 unique Entrez Genes**			
**Entrez Gene ID**	**Gene Symbol**	**Gene Name**	**NCBI Gene Function**
114	ADCY8	adenylate cyclase 8 (brain)	membrane bound enzyme that catalyses the formation of cyclic AMP from ATP
5099	PCDH7	protocadherin 7	The gene product is an integral membrane protein that is thought to function in cell-cell recognition and adhesion
7625	ZNF74	zinc finger protein 74	–
23078	VWA8	von Willebrand factor A domain containing 8	–
55591	VEZT	vezatin, adherens junctions transmembrane protein	This gene encodes a transmembrane protein which has been localized to adherens junctions and shown to bind to myosin VIIA
57521	RPTOR	regulatory associated protein of MTOR, complex 1	encodes a component of a signaling pathway that regulates cell growth in response to nutrient and insulin levels
79755	ZNF750	zinc finger protein 750	This gene encodes a protein with a nuclear localization site and a C2H2 zinc finger domain. Mutations in this gene have been associated with seborrhea-like dermatitis with psoriasiform elements.
80757	TMEM121	transmembrane protein 121	–
114784	CSMD2	CUB and Sushi multiple domains 2	–
122706	PSMB11	proteasome (prosome, macropain) subunit, beta type, 11	Proteasomes generate peptides that are presented by major histocompatibility complex (MHC) I molecules to other cells of the immune system.
254827	NAALADL2	N-acetylated alpha-linked acidic dipeptidase-like 2	–
338707	B4GALNT4	beta-1,4-N-acetyl-galactosaminyl transferase 4	–
388228	SBK1	SH3 domain binding kinase 1	–
440073	IQSEC. 3	IQ motif and Sec. 7 domain 3	–
100507290	ZNF865	zinc finger protein 865	–

**B) 11 persistently hypomethylated DMRs annotated to 9 unique Entrez Genes**			

**Entrez Gene ID**	**Gene Symbol**	**Gene Name**	**NCBI Gene Function**

290	ANPEP	alanyl (membrane) aminopeptidase	Aminopeptidase N is located in the small-intestinal and renal microvillar membrane, and also in other plasma membranes. In the small intestine aminopeptidase N plays a role in the final digestion of peptides generated from hydrolysis of proteins by gastric and pancreatic proteases.
29982	NRBF2	nuclear receptor binding factor 2	–
136051	ZNF786	zinc finger protein 786	–
414763	BMS1P18	BMS1 ribosome biogenesis factor pseudogene 18	–
643955	ZNF733P	zinc finger protein 733, pseudogene	–
646096	CHEK2P2	checkpoint kinase 2 pseudogene 2	–
647121	EMBP1	embigin pseudogene 1	–
100101266	HAVCR1P1	hepatitis A virus cellular receptor 1 pseudogene 1	–
101927554	LINC01250	long intergenic non-protein coding RNA 1250	–

**Table 2:** The ‘enriched pathway-based sets’ from the HOMER annotated genes of the 3006 hypermethylated DMRs and the 3077 hypomethylated DMRs after the exposure of PHHs for 5 days daily to VPA. PHHs, primary human hepatocytes; VPA, valproic acid; DMRs, differentially methylated DNA regions.

**Pathway name**	**Set size**	**Candidates, contained**	**p-value**	**q-value**	**Pathway source**
Developmental Biology	586	129 (22.0%)	3.23E-10	8.02E-07	Reactome
Axon guidance	459	101 (22.0%)	3.06E-08	3.81E-05	Reactome
Wnt signaling pathway - Homo sapiens (human)	140	36 (25.7%)	3.17E-05	0.0262	KEGG
Axon guidance - Homo sapiens (human)	127	33 (26.0%)	5.33E-05	0.0331	KEGG
Signalling by NGF	386	76 (19.7%)	9.86E-05	0.0447	Reactome
Signaling by SCF-KIT	264	56 (21.2%)	0.000108	0.0447	Reactome
Hippo signaling pathway - Homo sapiens (human)	154	36 (23.4%)	0.000257	0.0914	KEGG
Diseases of signal transduction	180	40 (22.2%)	0.000373	0.097	Reactome
Regulation of lipid metabolism by Peroxisome proliferator-activated receptor alpha (PPARalpha)	20	9 (45.0%)	0.00044	0.097	Reactome
Oxytocin signaling pathway - Homo sapiens (human)	159	36 (22.6%)	0.000494	0.097	KEGG
BMP2 signaling TGF-beta MV	56	17 (30.4%)	0.000501	0.097	INOH
Fc gamma R-mediated phagocytosis - Homo sapiens (human)	92	24 (26.1%)	0.000502	0.097	KEGG
effects of calcineurin in keratinocyte differentiation	13	7 (53.8%)	0.000508	0.097	BioCarta
NOTCH1 Intracellular Domain Regulates Transcription	47	15 (31.9%)	0.000587	0.104	Reactome
Calcium signaling pathway - Homo sapiens (human)	180	39 (21.7%)	0.000741	0.118	KEGG
Signaling by ERBB4	263	52 (19.8%)	0.00107	0.118	Reactome
Signaling by PDGF	301	58 (19.3%)	0.0011	0.118	Reactome
Signaling by FGFR3	270	53 (19.6%)	0.00114	0.118	Reactome
Signaling by FGFR4	270	53 (19.6%)	0.00114	0.118	Reactome
Signaling by FGFR1	271	53 (19.6%)	0.00125	0.118	Reactome
Extrinsic Pathway of Fibrin Clot Formation	5	4 (80.0%)	0.00126	0.118	Reactome
G alpha (12/13) signalling events	76	20 (26.3%)	0.00127	0.118	Reactome
alk in cardiac myocytes	27	10 (37.0%)	0.00134	0.118	BioCarta
TGF-beta super family signaling pathway canonical	115	27 (23.5%)	0.00135	0.118	INOH
nuclear receptors coordinate the activities of chromatin remodeling complexes and coactivators to facilitate initiation of transcription in carcinoma cells	8	5 (62.5%)	0.00145	0.118	BioCarta
Signaling by FGFR2	273	53 (19.4%)	0.00148	0.118	Reactome
Interleukin-3, 5 and GM-CSF signaling	211	43 (20.4%)	0.00152	0.118	Reactome
Downstream signaling of activated FGFR2	267	52 (19.5%)	0.00152	0.118	Reactome
Downstream signaling of activated FGFR1	267	52 (19.5%)	0.00152	0.118	Reactome
Downstream signaling of activated FGFR3	267	52 (19.5%)	0.00152	0.118	Reactome
Downstream signaling of activated FGFR4	267	52 (19.5%)	0.00152	0.118	Reactome
Signaling by FGFR	274	53 (19.3%)	0.00161	0.118	Reactome
Hypertrophic cardiomyopathy (HCM) - Homo sapiens (human)	83	21 (25.3%)	0.00167	0.118	KEGG
Signaling by Insulin receptor	262	51 (19.5%)	0.0017	0.118	Reactome
Androgen receptor signaling pathway	89	22 (24.7%)	0.00181	0.118	Wikipathways
Signaling by NOTCH1	73	19 (26.0%)	0.00191	0.118	Reactome
Ectoderm Differentiation	141	31 (22.0%)	0.00194	0.118	Wikipathways
Signaling by ERBB2	277	53 (19.1%)	0.00206	0.118	Reactome
Interleukin-2 signaling	202	41 (20.3%)	0.00209	0.118	Reactome
HTLV-I infection - Homo sapiens (human)	259	50 (19.3%)	0.00224	0.118	KEGG
Arrhythmogenic Right Ventricular Cardiomyopathy	74	19 (25.7%)	0.00227	0.118	Wikipathways
Arrhythmogenic right ventricular cardiomyopathy (ARVC) - Homo sapiens (human)	74	19 (25.7%)	0.00227	0.118	KEGG
Constitutive Signaling by NOTCH1 HD+PEST Domain Mutants	53	15 (28.3%)	0.0023	0.118	Reactome
Constitutive Signaling by NOTCH1 PEST Domain Mutants	53	15 (28.3%)	0.0023	0.118	Reactome
Signaling by NOTCH1 PEST Domain Mutants in Cancer	53	15 (28.3%)	0.0023	0.118	Reactome
Signaling by NOTCH1 HD+PEST Domain Mutants in Cancer	53	15 (28.3%)	0.0023	0.118	Reactome
Signaling by NOTCH1 in Cancer	53	15 (28.3%)	0.0023	0.118	Reactome
Platelet activation - Homo sapiens (human)	131	29 (22.1%)	0.00239	0.118	KEGG
Adipogenesis	131	29 (22.1%)	0.00239	0.118	Wikipathways
Downstream signal transduction	279	53 (19.0%)	0.00242	0.118	Reactome
Signaling by EGFR	292	55 (18.8%)	0.00246	0.118	Reactome
Regulation of nuclear beta catenin signaling and target gene transcription	80	20 (25.0%)	0.00248	0.118	PID
Validated nuclear estrogen receptor alpha network	64	17 (26.6%)	0.00257	0.118	PID
NCAM signaling for neurite out-growth	223	44 (19.7%)	0.0026	0.118	Reactome
Downstream signaling events of B Cell Receptor (BCR)	120	27 (22.5%)	0.0026	0.118	Reactome
BMP signaling Dro	34	11 (32.4%)	0.00275	0.122	INOH
Signaling by Leptin	193	39 (20.2%)	0.00287	0.124	Reactome
Inactivation of Cdc42 and Rac	9	5 (55.6%)	0.00291	0.124	Reactome
DAP12 signaling	282	53 (18.8%)	0.00306	0.129	Reactome
Mesodermal Commitment Pathway	76	19 (25.0%)	0.00314	0.129	Wikipathways
Insulin receptor signalling cascade	238	46 (19.3%)	0.00321	0.129	Reactome
regulation of pgc-1a	21	8 (38.1%)	0.00332	0.129	BioCarta
EPH-ephrin mediated repulsion of cells	30	10 (33.3%)	0.00332	0.129	Reactome
Retinoic acid receptors-mediated signaling	30	10 (33.3%)	0.00332	0.129	PID
FoxO signaling pathway - Homo sapiens (human)	134	29 (21.6%)	0.0034	0.13	KEGG
Interleukin receptor SHC signaling	195	39 (20.0%)	0.00346	0.13	Reactome
NGF signalling via TRKA from the plasma membrane	310	57 (18.4%)	0.00361	0.133	Reactome
NRAGE signals death through JNK	45	13 (28.9%)	0.00363	0.133	Reactome
Constitutive Signaling by Aberrant PI3K in Cancer	61	16 (26.2%)	0.00389	0.14	Reactome
AMPK signaling pathway - Homo sapiens (human)	124	27 (21.8%)	0.00422	0.148	KEGG
MAPK1/MAPK3 signaling	191	38 (19.9%)	0.00424	0.148	Reactome
Signaling by VEGF	274	51 (18.6%)	0.00444	0.149	Reactome
Wnt Signaling Pathway and Pluripotency	101	23 (22.8%)	0.00445	0.149	Wikipathways
VEGFR2 mediated cell proliferation	198	39 (19.7%)	0.00455	0.149	Reactome
FRS-mediated FGFR2 signaling	186	37 (19.9%)	0.00475	0.149	Reactome
FRS-mediated FGFR1 signaling	186	37 (19.9%)	0.00475	0.149	Reactome
FRS-mediated FGFR3 signaling	186	37 (19.9%)	0.00475	0.149	Reactome
FRS-mediated FGFR4 signaling	186	37 (19.9%)	0.00475	0.149	Reactome
Dilated cardiomyopathy - Homo sapiens (human)	90	21 (23.3%)	0.00475	0.149	KEGG
repression of WNT target genes	10	5 (50.0%)	0.00519	0.16	Reactome
MAPK family signaling cascades	225	43 (19.1%)	0.00529	0.16	Reactome
MAPK signaling pathway - Homo sapiens (human)	257	48 (18.7%)	0.00529	0.16	KEGG
transcription regulation by methyltransferase of carm1	14	6 (42.9%)	0.00556	0.16	BioCarta
Netrin-1 signaling	37	11 (29.7%)	0.0057	0.16	Reactome
IRS-related events triggered by IGF1R	239	45 (18.8%)	0.00583	0.16	Reactome
IGF1R signaling cascade	239	45 (18.8%)	0.00583	0.16	Reactome
Signaling by Type 1 Insulin-like Growth Factor 1 Receptor (IGF1R)	239	45 (18.8%)	0.00583	0.16	Reactome
Neuronal System	291	53 (18.2%)	0.00595	0.16	Reactome
DAP12 interactions	298	54 (18.1%)	0.00614	0.16	Reactome
Gastric acid secretion - Homo sapiens (human)	75	18 (24.0%)	0.00631	0.16	KEGG
Fatty acid, triacylglycerol, and ketone body metabolism	98	22 (22.4%)	0.00637	0.16	Reactome
PIP3 activates AKT signaling	98	22 (22.4%)	0.00637	0.16	Reactome
PI-3K cascade:FGFR2	98	22 (22.4%)	0.00637	0.16	Reactome
PI-3K cascade:FGFR1	98	22 (22.4%)	0.00637	0.16	Reactome
PI-3K cascade:FGFR3	98	22 (22.4%)	0.00637	0.16	Reactome
PI-3K cascade:FGFR4	98	22 (22.4%)	0.00637	0.16	Reactome
PI3K events in ERBB4 signaling	98	22 (22.4%)	0.00637	0.16	Reactome
PI3K events in ERBB2 signaling	98	22 (22.4%)	0.00637	0.16	Reactome
VEGFA-VEGFR2 Pathway	266	49 (18.4%)	0.00639	0.16	Reactome
Collagen biosynthesis and modifying enzymes	64	16 (25.0%)	0.00644	0.16	Reactome
IRS-mediated signalling	235	44 (18.7%)	0.00704	0.173	Reactome
O-linked glycosylation	105	23 (21.9%)	0.00731	0.173	Reactome
RAF/MAP kinase cascade	185	36 (19.5%)	0.00759	0.173	Reactome
SHC1 events in EGFR signaling	185	36 (19.5%)	0.00759	0.173	Reactome
SOS-mediated signalling	185	36 (19.5%)	0.00759	0.173	Reactome
GRB2 events in EGFR signaling	185	36 (19.5%)	0.00759	0.173	Reactome
SHC1 events in ERBB2 signaling	185	36 (19.5%)	0.00759	0.173	Reactome
SHC1 events in ERBB4 signaling	185	36 (19.5%)	0.00759	0.173	Reactome
GRB2 events in ERBB2 signaling	185	36 (19.5%)	0.00759	0.173	Reactome
Regulation of Commissural axon pathfinding by Slit and Robo	4	3 (75.0%)	0.00784	0.175	Reactome
Fosphenytoin (Antiarrhythmic) Metabolism Pathway	4	3 (75.0%)	0.00784	0.175	SMPDB
Ephrin B reverse signaling	24	8 (33.3%)	0.0084	0.186	PID
Signaling by Interleukins	270	49 (18.1%)	0.00851	0.186	Reactome
Transport of organic anions	11	5 (45.5%)	0.00852	0.186	Reactome
Rho GTPase cycle	125	26 (20.8%)	0.00914	0.194	Reactome
PI3K/AKT activation	101	22 (21.8%)	0.00918	0.194	Reactome
Thyroid hormone signaling pathway - Homo sapiens (human)	119	25 (21.0%)	0.0092	0.194	KEGG
Signaling by NOTCH	107	23 (21.5%)	0.00923	0.194	Reactome
Cell death signalling via NRAGE, NRIF and NADE	61	15 (24.6%)	0.0096	0.2	Reactome

The steatosis related pathways were shown in red.

**Table 3:** Names and FCs of the 33 neighbors of the EntrezGene ID 2033 (see Fig. 1) after the exposure of PHHs for 5 days daily to VPA. PHHs, primary human hepatocytes; VPA, valproic acid; FCs, fold changes.

**Entrez Gene ID**	**Gene Symbol**	**Gene Name**	**MeDIP-seq FCs**	**Steatosis-related pathway(s)**
354	KLK3	kallikrein-related peptidase 3	1.9	–
595	CCND1	cyclin D1	2.1	–
864	RUNX3	runt-related transcription factor 3	2	–
				
1026	CDKN1A	cyclin-dependent kinase inhibitor 1 A (p21, Cip1)	-3.5	Adipogenesis
				Signaling events mediated by HDAC Class III
				
1387	CREBBP	CREB binding protein	-3.2	Signaling events mediated by HDAC Class III
				Transcriptional regulation of white adipocyte differentiation
				Regulation of lipid metabolism by Peroxisome proliferator-activated receptor alpha (PPARalpha)
				Signaling events mediated by HDAC Class I
				Fatty acid, triacylglycerol, and ketone body metabolism
1488	CTBP2	C-terminal binding protein 2	-3.9	–
1844	DUSP2	dual specificity phosphatase 2	2.4	–
1958	EGR1	early growth response 1	-3.6	–
				
2033	EP300	E1A binding protein p300	-5.2	Signaling events mediated by HDAC Class III
				Transcriptional regulation of white adipocyte differentiation
				Signaling events mediated by HDAC Class I
2309	FOXO3	forkhead box O3	2.2	Signaling events mediated by HDAC Class III
2353	FOS	FBJ murine osteosarcoma viral oncogene homolog	-3.5	–
2626	GATA4	GATA binding protein 4	2.5	Adipogenesis
4088	SMAD3	SMAD family member 3	2.4	Adipogenesis
4205	MEF2A	myocyte enhancer factor 2 A	2.2	Adipogenesis
4772	NFATC1	nuclear factor of activated T-cells, cytoplasmic, calcineurin-dependent 1	1.8	–
5594	MAPK1	mitogen-activated protein kinase 1	2.3	–
5914	RARA	retinoic acid receptor, alpha	-3.8	Adipogenesis
6095	RORA	RAR-related orphan receptor A	-4	Adipogenesis
				
6256	RXRA	retinoid X receptor, alpha	-3.8	Adipogenesis
				Transcriptional regulation of white adipocyte differentiation
				Regulation of lipid metabolism by Peroxisome proliferator-activated receptor alpha (PPARalpha)
				Fatty acid, triacylglycerol, and ketone body metabolism
6670	SP3	Sp3 transcription factor	-4.1	–
6776	STAT5A	signal transducer and activator of transcription 5 A	2.2	Adipogenesis
6925	TCF4	transcription factor 4	-4	–
6929	TCF3	transcription factor 3	2.1	–
6934	TCF7L2	transcription factor 7-like 2 (T-cell specific, HMG-box)	-3.4	–
6938	TCF12	transcription factor 12	2.12	–
7026	NR2F2	nuclear receptor subfamily 2, group F, member 2	2.6	–
7161	TP73	tumor protein p73	2.5	–
9252	RPS6KA5	ribosomal protein S6 kinase, 90 kDa, polypeptide 5	-3.9	–
9314	9314	Kruppel-like factor 4 (gut)	2.7	–
				
9611	NCOR1	nuclear receptor corepressor 1	-3.4	Adipogenesis
				Transcriptional regulation of white adipocyte differentiation
				Regulation of lipid metabolism by Peroxisome proliferator-activated receptor alpha (PPARalpha)
				Signaling events mediated by HDAC Class I
				Fatty acid, triacylglycerol, and ketone body metabolism
10365	KLF2	Kruppel-like factor 2	-3.3	Signaling events mediated by HDAC Class III
23411	SIRT1	sirtuin 1	-3.5	
90427	BMF	Bcl2 modifying facto	2.2	

## 2 Experimental design, materials and methods

### 2.1 Cell culture and treatment

Cryopreserved primary human hepatocytes (PHHs, Invitrogen) of 3 individuals (Hu8084, Hu4197 and Hu4227) were thawed for 1 minute at 37 °C in a water bath. Next, these PHHs were pooled in order to bypass inter-individual variability in susceptibility to toxicants and cultured in 6-well plates in a collagen sandwich [2], according to the supplier's protocol (Invitrogen). After 3 days, the PHHs were daily exposed to 15 mM VPA or 1% EtOH (vehicle control) in culture medium for 5 days. Culture medium was changed daily thereby administering a new incubation concentration of VPA or EtOH to the cells. After the exposure period of 5 days, PHHs were lysed for DNA isolation. Another well of PHHs was maintained in culture for 3 consecutive days without VPA-treatment (called washout); the culture medium was again changed every day. All experiments were performed in biological triplicates.

### 2.2 DNA isolation

PHHs were collected in 500 μL of digestion buffer (1 mM EDTA; 50 mM Tris–HCl, pH 8.0; 5% SDS) and proteinase K (1 mg/ml) (Ambion). After incubation for 1 hour at 55 °C, the proteinase K was inactivated at 80 °C. RNAse A (400 μg/ml) (Qiagen) and 1% collagenase (Sigma) treatment was performed for 1 h at 37 °C. An equal amount of phenol-chloroform-isoamylalcohol (PCI; 25:24:1) (Sigma) was added and shaken manually for 5 minutes. After centrifugation, the upper phase was again treated with PCI until protein was no longer visible at the interphase. The upper phase was precipitated using 50 µL of 3 M NaAc pH 5.6 and 1250 µL of cold 100% ethanol. The DNA pellet was washed using cold 70% EtOH, dissolved in 50 µL of nuclease free water and quantified spectrophotometrically using the NanoDrop 1000 (Thermo Scientific, Waltham, MA). The total amount of DNA obtained was at least 10 µg of DNA, the 260/280 ratio laid between 1.7–1.9, and the 260/230 ratio was higher than 1.6.

### 2.3 MeDIP-seq protocol

MeDIP-seq was performed, with all the biological triplicates after DNA isolation, according to the protocol of Taiwo et al. [3], with minor adjustments.

#### 2.3.1 DNA fragmentation to a size of ~200 bp

For DNA fragmentation, 300 ng of isolated DNA were sonicated on the bioruptor (Diagenode) by using instrument settings of 15 cycles, each consisting of 30 seconds on/off periods. After fragmentation, the genomic DNA size range was assessed using an Agilent 2100 Bioanalyzer and high-sensitivity DNA chips (Agilent Technologies), according to the manufacturer's instructions.

#### 2.3.2 Library preparation and size selection

Libraries were prepared using 300 ng of fragmented DNA (~200 bp) and the NEBNext Ultra DNA Library Prep Kit for Illumina (NEB), according to the manufacturer's protocol.

#### 2.3.3 MeDIP analysis

The purified adaptor-ligated DNAs were used for Methylated DNA Immuno-Precipitation (MeDIP), according to the manufacturer's instructions of the MagMeDIP kit (Diagenode) and IPure kit (Diagenode).

#### 2.3.4 Quality control

Quantitative PCR (qPCR) was used for controlling DNA methylation enrichment. qPCR was performed by measuring the Ct-values of 1 µL of purified DNA sample and 24 µL of qPCR mixture (1 µL of provided primer pair (reverse and forward), 12.5 µL of SYBR Green PCR master mix and 10.5 µL water) using the temperature profile: 95 °C for 7 min, 40 cycles of 95 °C for 15 s. and 60 °C for 1 minute, followed by 1 minute 95 °C. The efficiency of MeDIP was calculated by performing qPCR and using the following formula: %(meDNA-IP/Total input) = 2^[(Ct(10%input)-3.32) – Ct(meDNA-IP)] × 100%. The efficiency for methylated DNA fragments was good (>50%) for all samples. More interestingly, the efficiency for non-methylated DNA fragments was overall lower than 1.0%.

#### 2.3.5 PCR amplification and size selection

PCR was used to amplify the MeDIP adaptor-ligated DNA fragments. In brief, 25 µL NEBNext High Fidelity 2x PCR Master mix (NEB), 1 µL of Index primer (NEB) that was used as a barcode for each sample, and 1 µL of Universal PCR primer (NEB) were added to 23 µL of the MeDIP adaptor ligated DNA fragments. PCR was performed by using the temperature profile: 98 °C for 30 s, 15 cycles of 98 °C for 10 s, 65 °C for 30 sec., and 72 °C for 30 s, followed by 5 minutes at 72 °C and hold on 4 °C as described before [3].

Thereafter, PCR-amplified DNAs (libraries) were cleaned using Cleanup of PCR Amplification in the NEBNext Ultra DNA Library Prep Kit for Illumina (NEB). Fragmented DNA size and quality were checked using the Agilent 2100 Bioanalyzer and high-sensitivity DNA chips (Agilent Technologies). In addition, generated libraries were size-selected on a 2% TAE low melting point (LMP) agarose gel; fragments of 250–350 bp were excised and the MinElute Gel DNA extraction kit (Qiagen) was used to extract and purify the DNA libraries. Libraries were quantified on a Qubit fluorimeter (Invitrogen) by using the Qubit dsDNA HS Assay kit (Invitrogen). All kits and chips were used according to the manufacturer's protocol.

#### 2.3.6 Sequencing

The 12 amplified libraries, each sample having its own index primer, were pooled at an equimolar concentration of 2 nM, based on Qubit measurements. Ten, 15, and 20 pM of the 2 nM stock solution were then loaded onto three separated channels of a 1.4 mm flow cell (Illumina) and cluster amplification was performed on a cBot (Illumina). Clusters were generated on cBot (Illumina) using the TruSeq® PE Cluster Kit V3, according to the manufacturer's instructions (Illumina), and the paired-end libraries were sequenced using 2 × 100 cycles TruSeq™ SBS Kit v3 paired-end by sequencing by synthesis (SBS) on the Illumina HiSeq. 2000. Base calling was performed by using Casava 1.8.2 (Illumina) and de-multiplexing by using bcl2fastq 1.8.4 (Illumina). Sequence reads were aligned against the human reference genome called UCSC hg19. This alignment produces FASTQ files for each barcoded library. MeDIP-seq raw data are available on ArrayExpress (accession number: E-MTAB-4437).

### 2.4 Data analysis

#### 2.4.1 MeDIP-Seq analysis

FastQC was applied to check the quality of the 100 bp reads pairs of the 12 sequenced samples. Paired-end sequencing reads were aligned against hg19 using Bowtie2 software. The MEDIPS package (version 1.16.0, Bioconductor) was used for the analysis of the MeDIP-seq data [4], [5], [6]. The default parameters described in the MEDIPS guideline (version 1.16.0) [7] were applied to all data from individual chromosomes, including mitochondrial DNA (chrM). The dataset was divided into four different groups of triplicates: (1) Control MeDIP samples includes the sequencing data of PHHs daily exposed during 5 days to the control vehicle; (2) VPA-treated MeDIP samples includes the sequencing data of PHHs exposed for 5 days daily to VPA; (3) Control washout MeDIP samples contains the sample exposed for 5 days daily with the vehicle control followed by a washout-period of 3 days; and (4) VPA-treated followed by washout MeDIP samples includes the sequencing data of PHHs exposed treated daily by VPA for 5 days followed by a washout-period of 3 days. Statistical analysis was performed applying the default parameters of MEDIPS, using the edgeR module, an empirical analysis of digital gene expression data in R that uses Bayes estimation and exact tests based on the negative binomial distribution [8]. Notably, raw count data was normalized using the weighted trimmed mean of M-values (TMM-normalization). Regions were considered significantly methylated if the edgeR.p-value was below 0.01 and if the number of reads, of a specific region, in one of the samples was higher than the mean of reads of all regions (the whole genome), which is the background correction. This p-value was derived from other studies performing MeDIP-seq analysis [9], [10], [11].

Annotation of DMRs into different genomic locations was achieved by using the HOMER software Regions were merged if (1) the start of a region was consecutive to the end of the previous region and (2) if the HOMER annotations of these consecutive DMRs were the same. Significant selected DMRs lists and unique gene lists were uploaded onto VENNY [12]. In this paper, the names and functions of the persistent genes are available in Table 1.

#### 2.4.2 Pathway analysis

ConsensusPathDB [13] was used to identify and visualize the involvement of the unique genes in biological processes that have been derived from affected pathways, by selecting significant pathways with a p-value < 0.01 from a gene enrichment analysis. In this paper, the significant pathways are available in Table 2.

#### 2.4.3 Network visualization

Methylated genes were then uploaded onto Cytoscape. The circular layout was selected and the network was analyzed as undirected. FCs were added and nodes were colored (green = hypermethylation (positive FCs) and red = hypomethylation (negative FCs)).

The first neighbors of methylated hub genes were selected by using the tool first neighbors of selected nodes in Cytoscape. Then, a sub-molecular induced epigenome network with its first neighbors was prepared in Cytoscape. In this publication, molecular interaction networks and a sub-molecular interaction network of the gene *EP300* is available in Fig. 1.

Furthermore, names, FCs, and presence of the gene in one or more steatosis related pathways of the 33 neighbors of EntrezGeneID 2033 (*EP300*) are available in Table 3.
